# A cancer-specific anti-podocalyxin monoclonal antibody (60-mG_2a_-f) exerts antitumor effects in mouse xenograft models of pancreatic carcinoma

**DOI:** 10.1016/j.bbrep.2020.100826

**Published:** 2020-10-10

**Authors:** Mika K. Kaneko, Tomokazu Ohishi, Manabu Kawada, Yukinari Kato

**Affiliations:** a Department of Antibody Drug Development, Tohoku University Graduate School of Medicine, 2-1 Seiryo-machi, Aoba-ku, Sendai, Miyagi, 980-8575, Japan; b Institute of Microbial Chemistry (BIKAKEN), Numazu, Microbial Chemistry Research Foundation, 18-24 Miyamoto, Numazu-shi, Shizuoka, 410-0301, Japan; c New Industry Creation Hatchery Center, Tohoku University, 2-1, Seiryo-machi, Aoba-ku, Sendai, Miyagi, 980-8575, Japan

**Keywords:** Podocalyxin, PODXL, Monoclonal antibody, Cancer-specific mAb, Pancreatic cancer, ADCC, antibody-dependent cellular cytotoxicity, BSA, bovine serum albumin, CasMab, cancer-specific monoclonal antibody, CBIS, Cell-Based Immunization and Screening, CDC, complement-dependent cytotoxicity, PBS, phosphate-buffered saline, PODXL, podocalyxin

## Abstract

Overexpression of podocalyxin (PODXL) is associated with progression, metastasis, and poor outcomes in several cancers. PODXL also plays an important role in the development of normal tissues. For antibody-based therapy to target PODXL-expressing cancers using monoclonal antibodies (mAbs), cancer-specificity is necessary to reduce the risk of adverse effects to normal tissues. In this study, we developed an anti-PODXL cancer-specific mAb (CasMab), named as PcMab-60 (IgM, kappa) by immunizing mice with soluble PODXL, which is overexpressed in LN229 glioblastoma cells. The PcMab-60 reacted with the PODXL-overexpressing LN229 (LN229/PODXL) cells and MIA PaCa-2 pancreatic cancer cells in flow cytometry but did not react with normal vascular endothelial cells (VECs), whereas one of non-CasMabs, PcMab-47 showed high reactivity for not only LN229/PODXL and MIA PaCa-2 cells but also VECs, indicating that PcMab-60 is a CasMab. Next, we engineered PcMab-60 into a mouse IgG_2a_-type mAb, named as 60-mG_2a_, to add antibody-dependent cellular cytotoxicity (ADCC). We further developed a core fucose-deficient type of 60-mG_2a_, named as 60-mG_2a_-f, to augment its ADCC activity. *In vivo* analysis revealed that 60-mG_2a_-f exerted antitumor activity in MIA PaCa-2 xenograft models at a dose of 100 μg/mouse/week administered three times. These results suggested that 60-mG_2a_-f could be useful for antibody-based therapy against PODXL-expressing pancreatic cancers.

## Highlights

• PODXL is associated with poor outcomes in several cancers.
• We developed an anti-PODXL cancer-specific mAb (PcMab-60).
• A core fucose-deficient IgG_2a_ type of PcMab-60 (60-mG_2a_-f) exerted antitumor activity in MIA PaCa-2 xenograft models.
• 60-mG_2a_-f could be useful for antibody-based therapy against PODXL-expressing pancreatic cancers.

## 1 Introduction

Podocalyxin (PODXL), also known as TRA-1-60 and TRA-1-81 antigens, is a highly *N*- or *O*-glycosylated type I transmembrane protein with a molecular weight of 150,000–200,000 [[1], [2], [3], [4]], and is used as pluripotent stem cell markers [3,[5], [6], [7], [8], [9]]. PODXL plays an important biological role in the kidney, heart, pancreatic, and breast tissues [10], and is also a prognostic indicator and a diagnostic marker for several malignant tumors such oral cancer [11], brain tumors [4], colorectal cancer [12], and renal cancer [13]. Because PODXL has been suggested to promote tumor growth, invasion, and metastasis [14,15], high PODXL expression could have adverse effects on overall survival (OS), disease-specific survival (DSS), and disease-free survival (DFS) in several cancers.

Despite the development of anti-PODXL monoclonal antibodies (mAbs), the efficacy of these treatments against cancers remains to be fully explained [5,16]. We previously immunized mice with recombinant soluble PODXL, which was purified from the culture supernatant of LN229/ectodomain-PODXL cells, and developed PcMab-47 of mouse IgG_1_ subclass [17]. We then produced a mouse-human chimeric mAb (chPcMab-47) and demonstrated its antitumor activity against colorectal cancers [18]. Furthermore, we established a chimeric anti-PODXL mAb (47-mG_2a_) by combining the variable region of PcMab-47 of mouse IgG_1_ and the constant region of mouse IgG_2a_ [19]. Moreover, we produced 47-mG_2a_-f, a core fucose-deficient 47-mG_2a_ to augment antibody-dependent cellular cytotoxicity (ADCC), and demonstrated high antitumor activity of 47-mG_2a_-f against oral cancers [19]. Although PcMab-47 and its modified mAbs are very useful for detecting PODXL in immunohistochemistry, they reacted with both cancer cells and normal cells, including vascular endothelial cells [[19], [20], [21], [22]]. Therefore, we could not transfer PcMab-47 into clinical use because its chimeric mAb or humanized mAb might show severe adverse effects.

For antibody-based therapy to target PODXL-expressing cancers, cancer-specificity is necessary to reduce the risk of adverse effects to normal tissues. In this study, we developed a cancer-specific anti-PODXL mAb, PcMab-60 (IgM, kappa) by immunizing mice with soluble PODXL. We engineered PcMab-60 into a mouse IgG_2a_-type mAb (60-mG_2a_) to add ADCC, and further produced a core fucose-deficient type of 60-mG_2a_ (60-mG_2a_-f) to augment its ADCC activity. We then examined the antitumor activity of 60-mG_2a_-f against a mouse xenograft of pancreatic cancer.

## 2 Materials and methods

### 2.1 Cell lines

The MIA PaCa-2 was obtained from the Cell Resource Center for the Biomedical Research Institute of Development, Aging and Cancer Tohoku University (Miyagi, Japan). P3U1 (mouse myeloma cell line) and LN229 (glioblastoma cell line) were obtained from the American Type Culture Collection (Manassas, VA). In our previous study (17), the LN229/PODXL and LN229/ectodomain-PODXL were produced. LN229, LN229/PODXL, LN229/ectodomain-PODXL, and MIA PaCa-2 were cultured in DMEM medium (Nacalai Tesque, Inc., Kyoto, Japan) and P3U1 was cultured in RPMI1640 medium (Nacalai Tesque, Inc.). The media were supplemented with 10% heat-inactivated fetal bovine serum (FBS; Thermo Fisher Scientific Inc., Waltham, MA), 100 units/ml of penicillin, 100 μg/mL of streptomycin, and 0.25 μg/mL of amphotericin B (Nacalai Tesque, Inc.). The Vascular endothelial cells-1 and Vascular endothelial cells-2 were purchased from Cambrex Corp. (Walkersville, MD), and were cultured in endothelial cell medium EGM-2MV, supplemented with 5% FBS (Cambrex Corp.). All cells were cultured at 37 °C in a humidified atmosphere containing 5% CO_2_ and 95% air.

### 2.2 Animals

All animal experiments were performed in accordance with relevant guidelines and regulations to minimize animal suffering and distress in the laboratory. The Animal Care and Use Committee of Tohoku University approved all the animal experiments for hybridoma production. Animal experiments for antitumor activity were approved by the Institutional Committee for Experiments of the Institute of Microbial Chemistry. Mice were maintained in a pathogen-free environment (23 ± 2 °C, 55 ± 5% humidity) on 11 h light/13 h dark cycle with food and water supplied *ad libitum* across the experimental period. Mice were monitored for health and weight every 2 or 5 days during the 3-week period of each experiment. The loss of original body weight to a point >25% and/or a maximum tumor size >3000 mm^3^ were identified as humane endpoints for euthanasia. Mice were euthanized by cervical dislocation; death was verified by respiratory and cardiac arrest.

### 2.3 Hybridoma production

We immunized four-week-old female BALB/c mice (CLEA, Tokyo, Japan) with the purified ectodomain of human PODXL (100 μg) together with Imject Alum (Thermo Fisher Scientific Inc.) by intraperitoneal (i.p.) injection. After several additional immunizations, a booster i.p. injection of LN229/PODXL was given 2 days before the mice were euthanized by cervical dislocation, and spleen cells were harvested. The spleen cells were fused with P3U1 cells using PEG1500 (Roche Diagnostics, Indianapolis, IN). Hybridomas were grown in RPMI 1640 medium including L-glutamine with hypoxanthine, aminopterin, and thymidine selection medium supplement (Thermo Fisher Scientific Inc.). Culture supernatants were screened using enzyme-linked immunosorbent assay (ELISA) for binding to the purified ectodomain of PODXL.

### 2.4 ELISA

Proteins were immobilized on Nunc Maxisorp 96-well immuno plates (Thermo Fisher Scientific, Inc.) at 1 μg/mL for 30 min. After blocking with 1% bovine-serum albumin (BSA) in 0.05% Tween 20/phosphate buffered saline (PBS, Nacalai Tesque, Inc.), the plates were incubated with culture supernatant followed by 1:2000 diluted peroxidase-conjugated anti-mouse immunoglobulins (Agilent Technologies, Inc., Santa Clara, CA). The enzymatic reaction was produced with a 1-Step Ultra TMB-ELISA (Thermo Fisher Scientific, Inc.). The optical density was measured at 655 nm using an iMark microplate reader (Bio-Rad Laboratories, Inc., Berkeley, CA).

### 2.5 Antibodies

PcMab-47, a mouse anti-PODXL mAb (IgG_1_, kappa), was developed as previously described [17]. The mouse IgG was purchased from Sigma-Aldrich Corp. (St. Louis, MO). To generate 60-mG_2a_, appropriate V_H_ cDNA of PcMab-60 and C_H_ of mouse IgG_2a_ were subcloned into pCAG-Ble vector (FUJIFILM Wako Pure Chemical Corporation, Osaka, Japan), and V_L_ and C_L_ cDNAs of PcMab-60 were subcloned into pCAG-Neo vector (FUJIFILM Wako Pure Chemical Corporation). To generate 60-mG_2a_, antibody expression vectors were transfected into ExpiCHO-S cells using the ExpiCHO Expression System (Thermo Fisher Scientific). To generate 60-mG_2a_-f, antibody expression vectors were also transfected into BINDS-09 (FUT8-knocked out ExpiCHO-S cells) using the ExpiCHO Expression System [23]. PcMab-60, 60-mG_2a_, and 60-mG_2a_-f were purified using Protein G-Sepharose (GE Healthcare Bio-Sciences, Pittsburgh, PA).

### 2.6 Flow cytometry

Cell lines were harvested via a brief exposure to 0.25% trypsin/1 mM ethylenediaminetetraacetic acid (EDTA; Nacalai Tesque, Inc.). After washing with 0.1% BSA in PBS, the cells were treated with 10 μg/mL of primary mAbs for 30 min at 4 °C, followed by treatment with Alexa Fluor 488-conjugated anti-mouse IgG (1:1000; Cell Signaling Technology, Danvers, MA). Fluorescence data were collected using an EC800 Cell Analyzer (Sony Corp., Tokyo, Japan).

### 2.7 Determination of binding affinity using flow cytometry

The MIA PaCa-2 cells (2 × 10^5^) were resuspended in 100 μL of serially diluted PcMab-60 and 60-mG_2a_-f (6 ng/mL to 100 μg/mL), followed by the addition of Alexa Fluor 488-conjugated anti-mouse IgG (1:200; Cell Signaling Technology). Fluorescence data were collected using a cell analyzer (EC800). The dissociation constant *K*_D_ was obtained by fitting the binding isotherms using the built-in one-site binding models in GraphPad PRISM 6 (GraphPad Software, La Jolla, CA).

### 2.8 Antitumor activity of anti-PODXL antibodies

Five-week-old female BALB/c nude mice were purchased from Charles River and used in experiments at 7 weeks of age. The cells (0.3 mL of 1.33 × 10^8^/mL in DMEM) were mixed with 0.5 mL of BD Matrigel Matrix Growth Factor Reduced (BD Biosciences, San Jose, CA). A 100-μL suspension (containing 5 × 10^6^ cells) was injected subcutaneously into the right flanks of nude mice. After 1 day, 100 μg of 60-mG_2a_-f or mouse IgG in 100 μL PBS were injected into the peritoneal cavity of each mouse. Additional antibodies were injected at days 8 and 15. The mice were euthanized 22 days after cell implantation. All data were expressed as the mean ± SEM. Statistical analysis was performed using the Tukey-Kramer test. *P* < 0.05 was considered statistically significant.

## 3 Results

### 3.1 Development of anti-PODXL mAbs

**Fig. 1:**
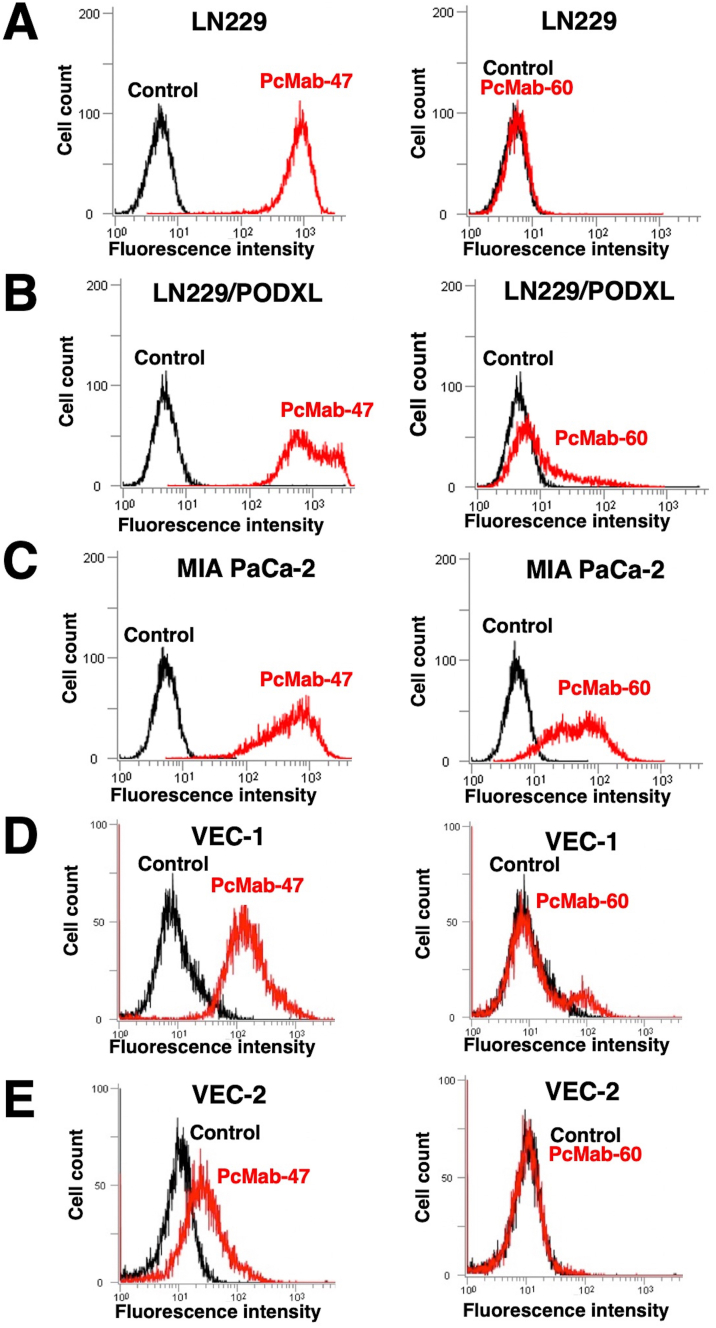
**Flow cytometry against cancer cells and normal cells using anti-PODXL mAbs (PcMab-47 and PcMab-60).** LN229 (A), LN229/PODXL (B), MIA PaCa-2 (C), VEC-1 (D), and VEC-2 (E) cells were treated with PcMab-47 (10 μg/mL), PcMab-60 (10 μg/mL), or control, followed by secondary antibodies. Vascular endothelial cells, VEC.

First, we immunized mice with recombinant PODXL, which was purified from the culture supernatant of LN229/ectodomain-PODXL. A booster i.p. injection of LN229/PODXL was administered. The culture supernatants were screened using ELISA for binding to purified PODXL. As a second screening, we performed flow cytometry for reaction with LN229 and LN229/PODXL, and a stronger reaction against LN229/PODXL than against LN229 was necessary. As a positive control, we used PcMab-47 [17], which strongly reacted with endogenous PODXL of LN229 glioblastoma cell line (Fig. 1A, left). The reaction of PcMab-47 to LN229/PODXL was slightly higher than that to LN229 (Fig. 1B, left). In this study, we developed a novel anti-PODXL mAb, PcMab-60 (IgM, kappa), which did not react with LN229 (Fig. 1A, right), but weakly reacted with LN229/PODXL (Fig. 1B, right). In contrast, PcMab-60 showed a higher reaction with MIA PaCa-2 pancreatic cancer cell line (Fig. 1C, right), although PcMab-47 reaction to MIA PaCa-2 is weaker than that of LN229 (Fig. 1C, left), indicating that PcMab-60 might be specific to pancreatic cancers. Because PODXL is highly expressed in vascular endothelial cells, PcMab-47 stained PODXL of two vascular endothelial cells (VEC-1 and VEC-2) as shown in Fig. 1D (left) and Fig. 1E (left). In contrast, PcMab-60 did not react with VEC-1 (Fig. 1D, right) and VEC-2 (Fig. 1E, right), indicating that PcMab-60 is cancer-specific.

### 3.2 Production of the mouse IgG_2a_-type antibody PcMab-60

**Fig. 2:**
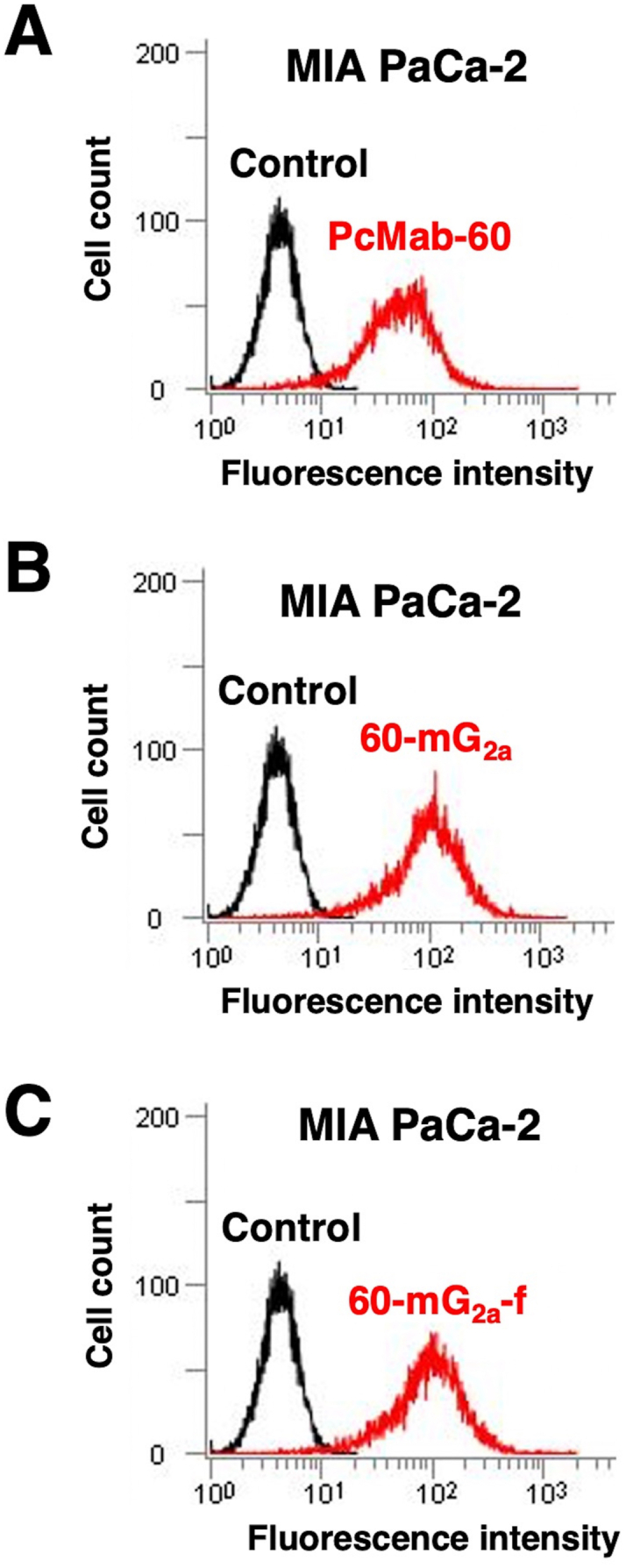
**Flow cytometry against MIA PaCa-2 cells using anti-PODXL mAbs (PcMab-60, 60-mG**_**2a**_**, and 60-mG**_**2a**_**-f).** MIA PaCa-2 cells were treated with PcMab-60 (10 μg/mL), 60-mG_2a_ (10 μg/mL), 60-mG_2a_-f (10 μg/mL), or control, followed by secondary antibodies.

Because the mouse IgG_2a_ possesses high ADCC and complement-dependent cytotoxicity (CDC) activities [24], we next developed IgG_2a_-type of PcMab-60, which was named as 60-mG_2a_. To generate 60-mG_2a_, appropriate V_H_ cDNA of PcMab-60 and C_H_ of mouse IgG_2a_ were subcloned into pCAG-Ble vector, and V_L_ and C_L_ cDNAs of PcMab-60 were subcloned into pCAG-Neo vector. Both vectors were transfected into ExpiCHO-S cells, and 60-mG_2a_ was purified from the supernatant. We further produced a core-fucose-deficient type of 60-mG_2a_, which was named as 60-mG_2a_-f using the BINDS-09 cell line (FUT8-knockout Expi–CHO–S cell line). Next, the reactivity of 60-mG_2a_ and 60-mG_2a_-f was confirmed by flow cytometry using MIA PaCa-2 cells. As shown in Fig. 2, PcMab-60, 60-mG_2a_, and 60-mG_2a_-f reacted strongly with MIA PaCa-2 cells.

### 3.3 The binding affinity of anti-PODXL mAbs

**Fig. 3:**
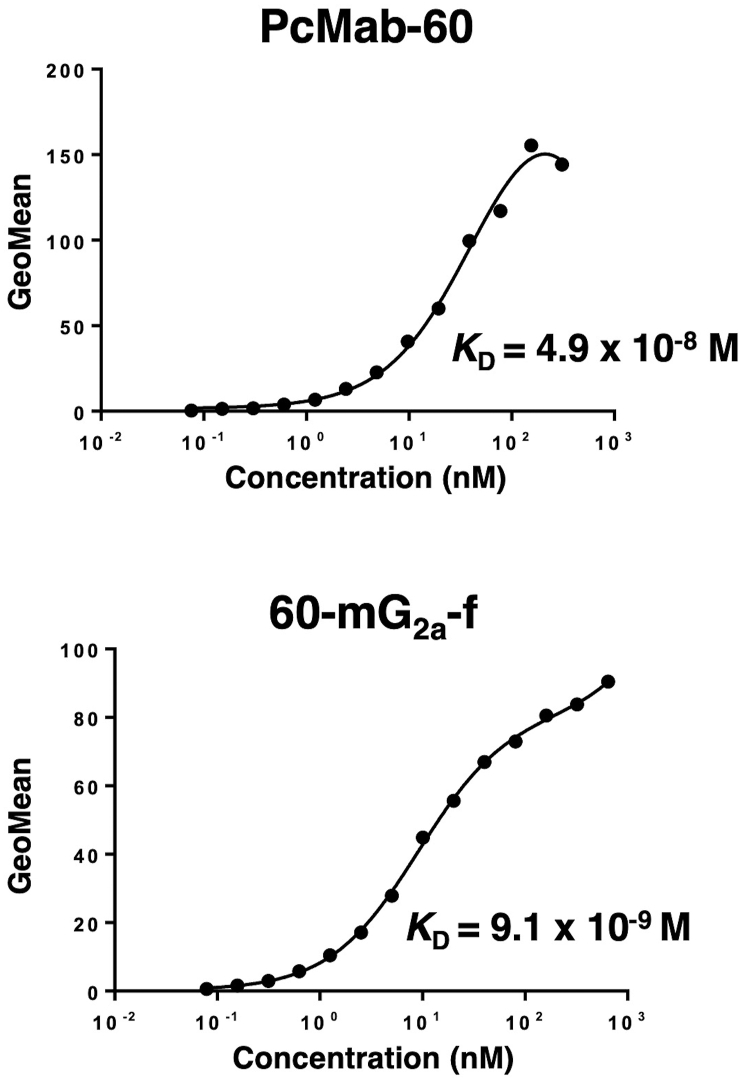
**Determination of binding affinity of anti-PODXL antibodies (PcMab-60 and 60-mG**_**2a**_**-f) using flow cytometry.** MIA PaCa-2 cells were suspended in 100 μL of serially diluted antibodies (6 ng/mL to 100 μg/mL), and secondary antibodies were then added. Fluorescence data were collected using a cell analyzer. GeoMean, the geometric mean of fluorescence intensity.

We performed a kinetic analysis of the interactions of PcMab-60 and 60-mG_2a_-f with MIA PaCa-2 cells using flow cytometry. As shown in Fig. 3, the dissociation constant (*K*_D_) for PcMab-60 against MIA PaCa-2 was 4.9 × 10^-8^ M. In contrast, the *K*_D_ for 60-mG_2a_-f was 9.1 × 10^−9^ M. The binding affinity of 60-mG_2a_-f against MIA PaCa-2 was 5.4-fold higher than that of PcMab-60. These results are compatible with our previous observations that all the chimeric antibodies, including chPcMab-47, 47-mG_2a_, and 47-mG_2a_-f, also possess a higher affinity for PODXL than the original PcMab-47 [19].

### 3.4 Antitumor activity of 60-mG_2a_-f against MIA PaCa-2 xenografts

**Fig. 4:**
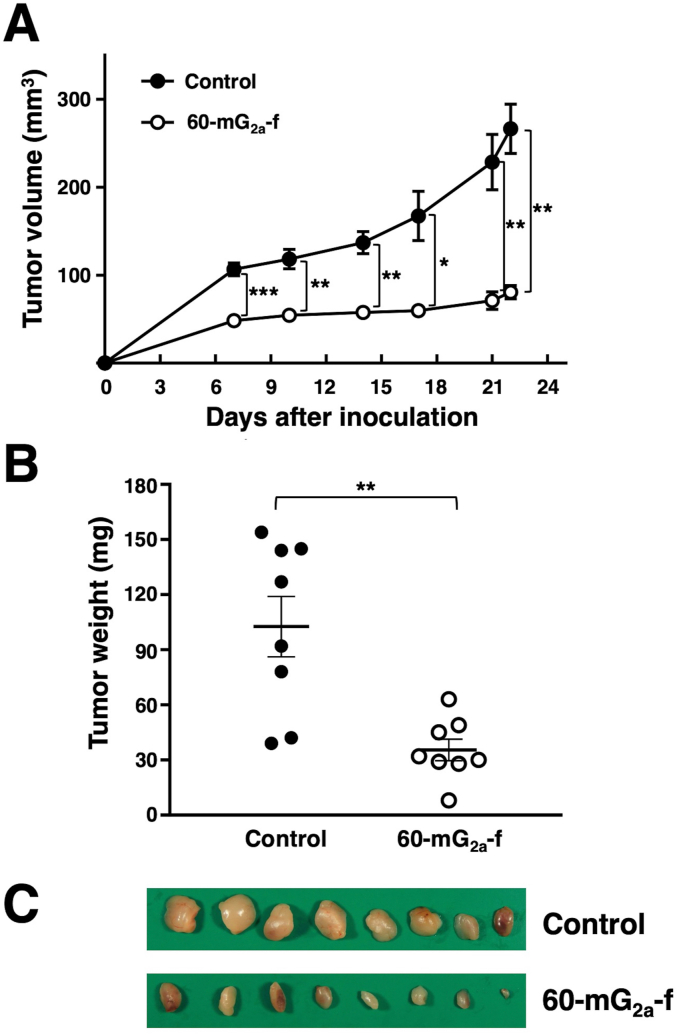
**Antitumor activity of 60-mG**_**2a**_**-f against MIA PaCa-2 xenograft.** (A) Tumor volume of MIA PaCa-2 xenografts. MIA PaCa-2 cells were injected subcutaneously into female nude mice into the 60-mG_2a_-f group (n = 8) and the control IgG group (n = 8). The indicated antibodies (100 μg/day; 5 mg/kg) were administered intraperitoneally 1, 8, and 15 days after cancer cell inoculation. The tumor volume was measured at the indicated time points. The values are means ± SEM. (B) Tumor weight of MIA PaCa-2 xenografts (day 22). The values are means ± SEM. An asterisk indicates statistical significance (*P < 0.05, **P < 0.01, ***P < 0.005, Tukey-Kramer's test). (C) Resected tumors of MIA PaCa-2 xenografts (day 22).

**Fig. 5:**
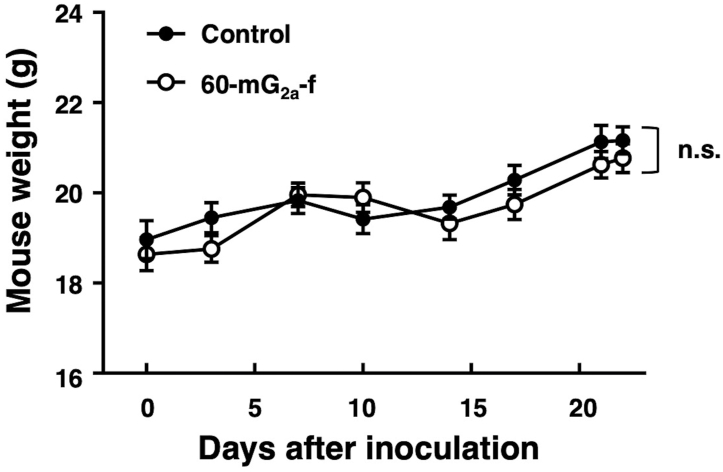
**Body weights of mice with MIA PaCa-2 xenografts.** MIA PaCa-2 cells were injected subcutaneously into female nude mice into the 60-mG_2a_-f group (n = 8) and the control IgG group (n = 8). The indicated antibodies (100 μg/day; 5 mg/kg) were administered intraperitoneally 1, 8, and 15 days after cancer cell inoculation. Body weights of mice with MIA PaCa-2 xenografts were measured at the indicated time points. The values are means ± SEM. n.s., not significant by Tukey-Kramer's test.

To investigate the antitumor effects of 60-mG_2a_-f on primary tumor growth *in vivo*, the MIA PaCa-2 cells were subcutaneously implanted into the flanks of nude mice. The 60-mG_2a_-f and the mouse IgG (control) were injected three times (100 μg of the antibodies on days 1, 8, and 15 after cell injections) into the peritoneal cavity of mice. Tumor formation was observed in all groups. The 60-mG_2a_-f significantly reduced tumor development compared with mouse IgG on days 7, 10, 14, 17, 21, and 22 (Fig. 4A). The tumor weights of mice in the 60-mG_2a_-f group were significantly lower than that in the IgG group on day 22 (Fig. 4B) The resected tumors of MIA PaCa-2 xenografts are depicted in Fig. 4C. Body weight was not significantly different between mouse IgG and 60-mG_2a_-f groups (Fig. 5).

## 4 Discussion

In our previous study, we developed the original technology for the production of cancer-specific monoclonal antibodies (CasMabs) against membrane proteins [[25], [26], [27]]. We have successfully produced anti-podoplanin CasMabs, such as LpMab-2 [25] and LpMab-23 [28] that specifically recognize cancer-type podoplanin in tumor tissues and not in normal-type podoplanin, which is expressed in lymphatic vessels. Moreover, the CasMab technology is useful for generating anti-glycopeptide mAbs (GpMabs). We have produced the following anti-podoplanin GpMabs: LpMab-3, LpMab-9, LpMab-12, LpMab-19, and LpMab-21 [[29], [30], [31]]. The other original technology for the production of sensitive and specific mAbs is the Cell-Based Immunization and Screening (CBIS) method [32]. We have developed several anti-podoplanin mAbs against pig [33], Tasmanian devil [34], alpaca [35], tiger [36], whale [37], goat [38], horse [39], and bear [40] PDPNs using the CBIS method.

In this study, we employed the CasMab technology, and successfully produced an anti-PODXL CasMab, PcMab-60, which highly reacts with MIA PaCa-2 pancreatic cell line, but did not react with PODXL-expressing vascular endothelial cells (Fig. 1). Although PcMab-60 was determined to be IgM, which usually shows low binding affinity, it showed moderate affinity (*K*_D_: 4.9 × 10^-8^ M) against MIA PaCa-2 cells (Fig. 3). Furthermore, 60-mG_2a_-f showed a higher binding affinity (*K*_D_: 9.1 × 10^-9^ M) compared with its original PcMab-60 (Fig. 3), indicating derivatives of PcMab-60, such as humanized mAbs of PcMab-60, could be useful for antibody-based therapy for pancreatic cancers. 60-mG_2a_-f and 60-mG_2a_ showed no difference in the binding affinity (data not shown). We have sometimes experienced that chimeric antibodies possess much higher affinity or lower affinity when compared with original mAbs [18,[41], [42], [43], [44], [45], [46]]. The stability of antibodies might be different among constant regions. We produced 60-mG_2a_-f, a non-fucosylated version of 60-mG_2a_ to augment its ADCC activities because non-fucosylated antibodies are known to show higher ADCC activities [47,48]. As expected, 60-mG_2a_-f exhibited high antitumor activities in a MIA PaCa-2 xenograft model (Fig. 4). In the future study, we need to combine PcMab-60 with anti-cancer drugs or include them in novel antitumor regimens, including T cells and viruses, to exert antitumor activity against cancer cells.

High PODXL expression was significantly associated with worse OS and was predictive of shorter OS in multiple cancers, especially pancreatic cancers [49]. It was also revealed that high PODXL expression predicted worse DSS and DFS. These results suggest that PODXL could be a prognostic factor, and diagnostic tools targeting this protein are expected. Unfortunately, we herein could not analyze PODXL expression in pancreatic cancer tissues because PcMab-60 was not useful for immunohistochemical analysis. In contrast, our previously established PcMab-47 was advantageous for immunohistochemical analysis [20]. The PODXL expression in pancreatic cancer tissues could be analyzed using PcMab-47, and then PODXL-positive patients should be treated using cancer-specific humanized PcMab-60.

## Declaration of competing interest

The authors declare no conflicts of interest involving this article.
